# The Nuclear Receptor PXR in Chronic Liver Disease

**DOI:** 10.3390/cells11010061

**Published:** 2021-12-27

**Authors:** Katia Sayaf, Ilaria Zanotto, Francesco Paolo Russo, Daniela Gabbia, Sara De Martin

**Affiliations:** 1 Department of Surgery, Oncology and Gastroenterology, University of Padova, 35121 Padova, Italy; katia.sayaf@phd.unipd.it (K.S.); francescopaolo.russo@unipd.it (F.P.R.); 2 Department of Pharmaceutical and Pharmacological Sciences, University of Padova, 35121 Padova, Italy; ilaria.zanotto1@studenti.unipd.it (I.Z.); sara.demartin@unipd.it (S.D.M.)

**Keywords:** nuclear receptors, pregnane X receptor, liver disease, fibrosis, liver cancer, cholestasis, NAFLD, NASH

## Abstract

Pregnane X receptor (PXR), a nuclear receptor known for modulating the transcription of drug metabolizing enzymes and transporters (DMETs), such as cytochrome P450 3A4 and P-glycoprotein, is functionally involved in chronic liver diseases of different etiologies. Furthermore, PXR activity relates to that of other NRs, such as constitutive androstane receptor (CAR), through a crosstalk that in turn orchestrates a complex network of responses. Thus, besides regulating DMETs, PXR signaling is involved in both liver damage progression and repair and in the neoplastic transition to hepatocellular carcinoma. We here summarize the present knowledge about PXR expression and function in chronic liver diseases characterized by different etiologies and clinical outcome, focusing on the molecular pathways involved in PXR activity. Although many molecular details of these finely tuned networks still need to be fully understood, we conclude that PXR and its modulation could represent a promising pharmacological target for the identification of novel therapeutical approaches to chronic liver diseases.

## 1. Introduction

Nuclear receptors (NRs) are components of a well-known superfamily of transcription factors involved in the modulation of different biological processes, including inflammation, apoptosis, cell cycle arrest, and drug metabolism [1,2]. In humans, the 48 members of this superfamily are classified in seven subfamilies (NR0-NR6), based on their mechanism of action [3]. Even though the 31% of NRs are considered “orphan” receptors, meaning that their ligands have not been identified yet, the main part of these receptors are regulated by well-known endogenous substances and/or xenobiotics [2,3]. Endogenous ligands for NRs are lipophilic hormones able to enter the cell. Some examples of these endocrine NRs are provided by the thyroid hormone receptors (TRα and TRβ) and the androgen (AR) and estrogen (ERα and ERβ) receptors [2,4].

The pregnane X receptor (PXR) belongs to the 1I subfamily and is a ligand-activated transcription factor, the classification of which is still controversial because a number of scientists still consider PXR an orphan receptor, although many ligands have been notified, since it binds a variety of endogenous compounds and xenobiotics at high (micromolar) concentrations [4,5]. However, regardless of its classification, PXR plays a key role in regulating hepatic homeostasis and responses to drugs, functioning as a so-called *xenosensor*, being activated by toxic compounds, thereby helping detoxification [6,7]. In the liver, PXR is highly expressed by hepatocytes, but its expression has been detected also in Kupffer and hepatic stellate cells (HSCs) [8,9,10,11]. Moreover, it has been reported that hepatic PXR expression is variable during development. Indeed, being low during fetal and neonatal stages, PXR expression in the liver increases during infancy, reaches a peak at puberty, and then decreases to fetal levels in the elderly [12]. Even though PXR expression seems to be not essential for development and its genetic deletion does not affect phenotypic traits, its hepatic activity and regulation have been related to the pathophysiology of many liver diseases.

This review summarizes the latest findings about PXR expression and its role in the hepatic environment, focusing on its connection to chronic liver diseases of different etiologies and underlining the inconsistencies in the results that have sometimes been obtained by studies conducted with different approaches. In the last part, a focus on gender-related differences in PXR activation and their relationship with disease development is reported.

## 2. PXR Structure, Expression, and Activation

PXR is also named NR1I2 (nuclear receptor subfamily 1 group I member 2) and SXR (steroid and xenobiotic receptor) since it is activated by steroid-like molecules, such as pregnane 21-carbon steroids [1]. PXR is predominantly located in the gut and in the liver, where it exerts its main role of modulating the transcription of hepatic genes encoding for phase I–II drug metabolizing enzymes (CYP3A, CYP2B, UDP-glucuronosyltransferases, sulfotransferase) and drug transporters, such as multidrug resistance proteins (MDRs) and multidrug resistance-associated proteins (MRPs) [7,13,14]. In the human liver, more than 15 splicing and transcript variants of PXR have been observed and correlated to alterations in its transcriptional activity. The predominant isoforms found in human liver are PXR1, which is the full-length isoform, and PXR2 and PXR3. PXR2 represents 6–15% of hepatic mRNA transcripts, whereas PXR3 is poorly expressed, representing 0–0.84% of all human PXR transcripts [15]. PXR variability has been shown to contribute to the interindividual variability in the expression of drug metabolizing enzymes and transporters (DMETs) [12]. For example, a small PXR variant (*sPXR*), encoding for a 37 kDa dominant negative isoform, is able to repress the function of the full-length PXR1 by competing with cofactors (e.g., SR1) for binding [16]. An inter-species variability has also been described. Two PXR isoforms, PXR1 and PXR2, are expressed in mice, the latter displaying a lower ligand activation profile with respect to PXR1 and directly repressing PXR1 activity [17].

**Figure 1:**
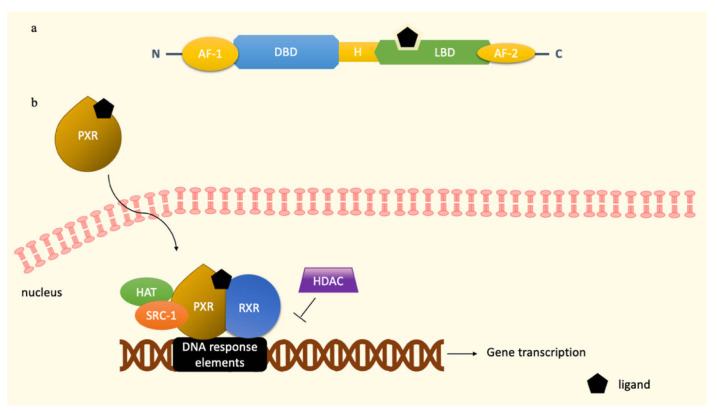
PXR structure (**a**) and activation following ligand binding (**b**). Upon ligand binding, PXR enters the nucleus and forms a complex with RXR. The recruitment of the coactivators HAT and SRC-1 into the complex further promotes PXR binding to DNA response elements, allowing the entrance of RNA polymerase II that starts gene transcription. HDAC downregulates PXR transcriptional activity. Abbreviations: DNA-binding domain (DBD), ligand-binding region (LBD), steroid receptor coactivator-1 (SRC-1), retinoid X receptor (RXR), histone acetyltransferase (HAT), histone deacetylase (HDAC).

As in other NRs, PXR is endowed with three domains, i.e., the N-terminus ligand-independent transactivation domains (AF-1), the DNA-binding domain (DBD), and a ligand-binding region (LBD) at the C-terminus, which is also responsible for the heterodimerization with retinoid X receptor (RXR) (Figure 1a). An additional ligand-dependent transactivation domain, named activation function 2 (AF-2), is located within the ligand binding domain [1,5,18]. Beyond the presence of PXR splicing and transcript variants, the finely tuned regulation of PXR transcriptional activity is due to the great variety of its ligands, including endobiotic compounds (pregnane, steroids, bile acids, vitamins) and xenobiotics (macrolide antibiotics, antifungals, toxins, and environmental pollutants) [4,14]. The capability of recognizing and binding a wide range of compounds is probably due to its structure, in particular to the great flexibility of its LBD [1]. As for other NRs, the first event leading to PXR activation consists in the binding of the ligand to the receptor LBD [19]. This binding site, normally characterized by an “α-helical sandwich”, has a unique five-stranded β-sheet that acts as a homodimerization site, forming tryptophan-zipper interactions [1,18]. Mutations of specific tryptophan residues (Trp223 and 225) within the structure of the PXR homodimer interface reduces its ability to interact with the steroid receptor coactivator-1 (SRC-1), leading to an impaired transcriptional activity of PXR [18,19]. The flexible and large ligand-binding pocket of PXR (from 1000 Å to 1540 Å, after ligand binding) is characterized by four distinct regions, i.e., a hydrophobic cage, a polar margin, a hydrophobic portion, and a polar duct [7,18,20]. As stated before, xenobiotics could take advantage of LBD flexibility to interact with the PXR receptor. For example, rifampicin interacts with 18 residues of the LBD, forming hydrogen bonds with Ser247, His407, and Gln285, while the HIV reverse transcriptase inhibitor PNU-142721 uses the hydrophobic cage to connect with PXR, through a π–π bond with Phe288 [18]. Some species-related differences observed in the affinity of PXR ligands could be ascribable to the low homology (73%) of the ligand-binding pocket of PXR between human and mouse [21]. Consequently, rifampicin shows a high affinity towards human but not murine PXR, while PCN is a potent activator of PXR in mice.

Two mechanisms have been proposed for PXR activation, i.e., the indirect and the direct mechanisms [4]. Although the “ligand-binding model” (direct) is the most popular, a non-classical (indirect) model of PXR activation has been recently investigated. This is based on the fact that several signaling pathways might alter the phosphorylation of PXR and/or associated proteins, leading to modifications of its activation [4]. For instance, in vivo studies assessed that forskolin triggers protein kinase A (PKA) signaling, enhancing the PXR-mediated induction of CYP3A4 in hepatocytes, upon recruitment of peroxisome proliferator-activated receptor gamma (PPARγ) coactivator-1 [4,19]. PKA signaling is also responsible for the binding between the coactivator SRC-1 and PXR. On the other hand, protein kinase C (PKC) signaling recruits corepressors, such as the nuclear receptor corepressor 1 (Ncor1), thereby inhibiting PXR activity [22].

By contrast, the direct mechanism of action is well-known and widespread among NRs and consists in the translocation of cytoplasmic PXR to the nucleus induced by ligand binding [23]. Upon ligand binding, the transactivation domains AF-1 and AF-2 act as interfaces of PXR since they mediate the recruitment of coregulators on specific target genes [5]. Interestingly, AF-2 plays a key role in defining the fate of PXR ligands as agonists or antagonists. When the AF-2 helix is placed in an “inward” spot, a compound is defined as an agonist, while an “outward” status of the helix imparts to the ligand an antagonist function [18]. Once in the nucleus, the ligand–PXR complex first binds to the retinoid X receptor α (RXRα), forming an heterotetrametric complex with the coactivator SRC-1 (Figure 1b), thereby enhancing the binding of the highly conserved zinc-fingers motifs present in the DBD to multiple DNA response elements in the promoter region of target genes [4,18,19]. Coactivators further recruit secondary coactivators and chromatin-associated enzyme, such as histone acetyltransferase (HAT), forming a multiprotein coactivator complex [24]. This prototypical transcriptional complex reorganizes the chromatin of the target genes, allowing the entrance of the transcriptional components of RNA polymerase II [7,23]. Conversely, the recruitment of histone deacetylases (HDACs) by corepressors downregulates gene transcription [7].

**Figure 2:**
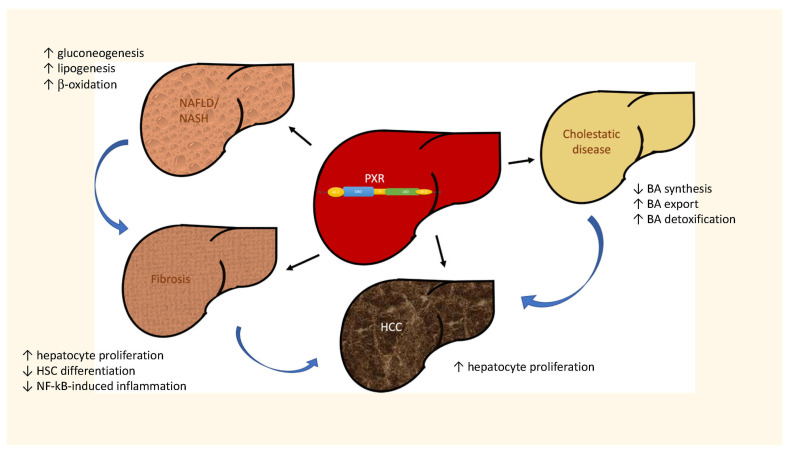
Role of PXR in chronic liver diseases of different etiology.

Upon binding to DNA response elements, PXR transcriptional activity regulates the expression of DMETs, e.g., CYP3A4, CYP2B, glutathione S-transferase, OATP2, and MDR1 [19,25]. Therefore, PXR plays a role in the regulation of many physiological and pathological processes in the liver, such as the metabolism of drugs, bile acids, and cholesterol, but has also been involved in molecular pathways triggering inflammation and cancer [19,25]. Hence, since PXR is responsible for controlling the transcription of genes involved in fibrogenesis, inflammation, and cell proliferation, many efforts have been addressed to unravel its role in the development of chronic liver diseases, a broad spectrum of different dysfunctions variable both for etiologies and pathological features. Several pieces of evidence of PXR regulation and activity have been obtained in these contexts, although sometimes controversial and disease specific. The findings on the different ways that PXR is regulated in the most common chronic liver diseases are summarized in Figure 2 and described in detail below.

## 3. PXR and Cholestasis

Cholestatic liver diseases include hereditary and acquired diseases causing the typical signs of an accumulation of cytotoxic bile acids (BAs), e.g., pruritus, and liver osteodystrophy [26,27]. The data obtained about the expression and putative role of PXR in cholestatic diseases are described below and will probably deserve further investigation since controversial evidence has been obtained so far. For example, clinical studies demonstrated that a marked increase in PXR gene and protein levels was observed in patients with obstructive cholestasis with respect to controls [28,29], whereas children with biliary atresia showed a PXR downregulation in the late stage of obstructive cholestasis [30].

### 3.1. The Homeostasis of Bile Acids and PXR

The liver is involved in the synthesis and transport of BAs [31], which are the result of cholesterol metabolism and act as emulsifiers to facilitate the absorption of liposoluble vitamins and lipids in the intestine, due to their amphiphilic nature [32,33]. A dysfunction of their synthesis in hepatocytes, an impairment or obstruction of bile flow or atypical bile secretion in proximity of cholangiocytes may induce cholestasis [32]. There are two main metabolic pathways for the synthesis of BAs, called either the *classical* (neutral) or *alternative* (acid) pathway [34]. Most of the BAs (about 75%) are produced by the former, including the primary BAs cholic acid (CA) and chenodeoxycholic acid (CDCA) [35]. This pathway starts with a 7α-hydroxylation of cholesterol by the rate limiting-enzyme CYP7A1, followed by the cleavage of the cholesterol side chain operated by CYP8B1, which determines the CA/CDCA ratio [34,36]. Interestingly, preclinical in vivo studies demonstrated that the genetic deletion of CYP7A1 caused a high perinatal mortality and severe malnourishment of liposoluble vitamins and lipids in mice that survived. However, it has also been observed that surviving mice were able to produce BAs, although in low amount, thereby suggesting the presence of an alternative pathway [37]. Other studies demonstrated that this alternative pathway is mainly extra-hepatic and starts with a sterol 27-hydroxylation of cholesterol catalyzed by CYP27A1, followed by a 7-α hydroxylation mediated by CYP7B1 producing three oxysterols [35,37,38]. The main outcome of the rate-limiting enzyme CYP7B1 is CDCA [34]. Once synthetized and before entering the bile canaliculi, the primary BAs are conjugated with glycine (in humans) and taurine (in humans and mice), released from hepatocytes, and stored in the gallbladder [32]. After meals, they are released into the small intestine to help the uptake of dietary lipids [39]. Once in the bowel, the gut microbiota is responsible for the deconjugation of primary BAs by the enzyme bile salt hydrolase (BSH) [34]. This reaction is followed by 7α-dehydroxylation and epimerization, leading the conversion of primary unconjugated into secondary conjugated BAs, such as lithocholic acid, deoxycholic acid, and ursodeoxycholic acid (UDCA) [31,32]. The 95% of BAs that are reabsorbed in the ileum by the enterohepatic cycle reach back to the liver [34]. Although a small portion of them passively cross the membrane of enterocytes, most of the BAs are reabsorbed by active transport by the apical sodium-dependent transporter (ASBT) [39]. Thanks to the basolateral organic solute transporter αβ (OSTαβ), they enter the portal circulation and cross the hepatocyte membrane via the organic anion-transporting polypeptide (OATP) or the sodium-taurocholate cotransporting polypeptide (NTCP) [31,39]. The remaining 5% is not absorbed in the ileum but reaches the colon, where the gut microbiota perform their deamination and dehydroxylation, allowing their passive reabsorption through the colonic epithelium. The BAs that are not reabsorbed by colon cells are excreted by feces [32,39]. The complexity of these mechanisms, i.e., synthesis, conjugation, uptake, and export of BAs, explains the reason why their homeostasis needs to be finely regulated by several cellular pathways, including PXR-mediated signaling.

A role of PXR in BA homeostasis was reported for the first time by Staudinger and colleagues, who demonstrated that lithocholic acid and its metabolites could act as PXR activators [32,40]. Nevertheless, some controversial findings have been reported so far regarding the concentration of BAs able to trigger PXR-mediated signaling. Indeed, some studies suggested that physiological concentrations of BAs are not sufficient to activate PXR-related pathways, whereas the increase in BA release occurring after a scratch damage of intrahepatic bile ducts does activate PXR, affecting the transcription of PXR-regulated genes, as for example CYP3A4, that are also responsible for BA detoxification [33,41]. Other PXR-regulated genes including enzymes, growth factors, bile acids transporters, and efflux pumps demonstrated a protective role towards cholestatic injury. Therefore, PXR seems to play an anti-cholestatic role by both inhibiting BA synthesis and enhancing the activity of the detoxification apparatus responsible for their elimination [32]. Interestingly, the homeostasis of BAs is regulated by the negative feedback of farnesoid X receptor (FXR), which upon BA binding activates a signaling cascade able to upregulate the enteral fibroblast growth factor 19 (FGF-19) [31]. Once in the hepatocyte, FGF-19 binds to the FGF receptor 4 (FGFR4), triggering the co-receptor β-klotho, which reduces the synthesis of BAs by inhibiting CYP7A1 transcription [42]. It has been demonstrated that PXR uses the FGF-19/CYP7A1 axis to help FXR in the homeostasis of BAs [32]. Although some evidence indicates that PXR expression increased in patients with cholestatic diseases (see below), further analyses on late-stage cholestasis revealed a downregulation of PXR [12]. This observation confirms the results indicating that PXR expression varies according to the different stages of cholestasis. Indeed, a preclinical in vivo study investigating PXR and CAR-mediated CYP3A modulation in bile duct ligated (BDL) rats showed that, after an initial upregulation during the first stages, these two NRs virtually disappeared in the late stages of cholestatic disease [43].

### 3.2. PXR and Autoimmune Cholestatic Diseases

The most diagnosed chronic cholestatic diseases are primary biliary cholangitis (PBC) and primary sclerosing cholangitis (PSC), both characterized by an immune-mediated damage affecting the small intrahepatic bile duct (PBC) or the intra- and extrahepatic large bile ducts (PSC) [32]. In addition to the immune-mediated damage, the pathogenic features of PBC and PSC include inflammation induced by BA toxicity and subsequent wound-healing responses with peculiar fibrotic outcomes [27,39].

PBC is a rare condition that predominantly affects women with a 10:1 ratio to males. Genetic factors, such as the above-stated female sex or variants in the human leukocyte antigens (HLA) locus, are known to influence the development of this disease [27,39]. Interestingly, the immune attack to biliary epithelial cells (BECs) is due to a loss of tolerance towards the modified E2 subunit of the mitochondrial pyruvate dehydrogenase complex (PDC-E2). This complex is recognized by antimitochondrial antibodies (AMAs) that, together with an infiltration of CD4+ and CD8+T cells, constitute the main pathogenic features of PBC [27,39]. Aberrant modifications of the PDC–E2 complex are responsible for the loss of BECs integrity. In addition, an impairment of anion exchanger 2 (AE2) activity on BECs membrane could alter the pH of cholangiocytes, creating an alkaline intracellular environment triggering cyclases able to acidify bile salts [39,44]. Consequently, BECs or cholangiocytes undergo apoptotic processes since they are sensitive to the action of cytotoxic BAs [39].

PSC is a rare condition affecting EU habitants with a ratio of 1:2000 [45]. Fibrosis and inflammation are responsible for multifocal biliary strictures, orchestrated by hepatic stellate cells, portal macrophages, and cholangiocytes [45]. Although the pathological mechanisms behind its development are not completely understood, there is a general consensus that other autoimmune diseases—for example, inflammatory bowel diseases (IBD)—represent a risk factor for PSC, and the senescence of cholangiocytes is directly associated to its severity [27,46].

Much evidence demonstrated that PXR expression is modulated in both PBC and PSC (Table 1). While alterations in PXR gene expression are known to play a significant role in the prognosis of PSC, a study by Poupon and collaborators demonstrated that the genetic background of PXR, specifically a single nucleotide polymorphism (SNP) C/A in exon 9 of 3′-UTR of NR1L2/PXR, is weakly correlated with the progression and severity of PBC [47]. Furthermore, it has been demonstrated that in PBC patients grade III and IV, PXR expression is reduced by 40–60% with respect to healthy subjects [48]. A study investigating the use of bezafibrate, a dual PPAR/PXR agonist, in PBC patients found a significant increase in PXR gene expression after treatment and suggested that this decrease may contribute to the anticholestatic activity of bezafibrate [49].

The common trait of PBC and PSC is the loss of tolerance against self-molecules, thus triggering an increased activation of the immune system and production of pro-inflammatory mediators, sometimes accompanied by extrahepatic autoimmune manifestations [50]. The increased amount of toxic BAs characterizing these diseases leads to the injury of cholangiocytes and activates a signaling cascade that sustains the release of pro-inflammatory cytokines and chemokines [51,52]. Notably, PBC patients show an elevated number of toll-like receptors (TLRs) on BECs. Upon TLR–ligand binding, a recruitment of leukocytes to the non-immune tolerated area on cholangiocytes occurs, probably AE2 or the PDC–E2 complex [52]. The stimulation of TLR-mediated signaling leads to the recruitment of pro-inflammatory factors, such as NF-kB factor and related cytokines [52]. Although the relationship between PXR and aberrant TLR-mediated immune responses in PBC is far from being completely understood, PXR has been proposed as a novel therapeutic target for diseases characterized by excessive TLR4 activation, such as intestinal inflammatory disorders, since PXR negatively regulates TLR4 [53]. It has been demonstrated that the PXR/RXR heterocomplex downregulates TLR4 signaling cascades and decreases inflammation. However, further studies aiming at understanding the involvement of the PXR–TLR4 pathway in the recruitment of immune cells should be performed.

The PXR activator rifampicin is used for the treatment of itches in cholestatic patients, and also for symptomatic gallstone disease [39,54]. Studies on rifampicin-treated patients undergoing cholecystectomy suggested that the therapeutic effect of the drug is due to the upregulation of the phase II uridine diphosphate glucuronosyltransferase (UGT1A1) and the multidrug resistance protein 2 transporter (MRP2), both mediated by PXR activation [41,55,56]. PXR activation has also been shown to upregulate the phase II enzyme sulfotransferase 2A1 (SULT2A1) in cholestatic disorders [32]. Based on Wunsch and colleagues’ findings, only in PBC patients was the overexpression of PXR in line with the enhanced transcriptional activity of SULT2A1, whereas PXR activation seems not to be responsible for increased levels of SULT2A1 in PSC patients, suggesting a disease-specific transcriptional effect of PXR on this enzyme [57].

## 4. PXR and Liver Steatosis

Nonalcoholic fatty liver disease (NAFLD) comprises a variable degree of liver dysfunction ranging from simple steatosis, where lipid droplets accumulate within hepatocytes, to nonalcoholic steatohepatitis (NASH), characterized by hepatic inflammation and fibrosis [58,59]. NAFLD has become a public health issue of increasing relevance, due to incorrect lifestyle and food habits, characterized by the excessive intake of fat and sugar [60]. Being usually not accompanied by visible pathological signs, NAFLD is hardly diagnosed in the initial phase, whereas when reaching the final stage of NASH it could decline to liver cirrhosis and hepatocellular carcinoma [59]. One common feature of liver steatosis of all degrees is the accumulation of lipids in the cytoplasm of hepatocytes, due to an impaired modulation of lipid intake, de novo lipogenesis, and lipid metabolism [36].

Since PXR is involved in the regulation of lipid metabolism, some studies have focused on investigating its role in NAFLD and NASH (Table 2). Although some experimental findings demonstrated a correlation between NAFLD and the dysregulation of genes controlled by PXR [61], this topic is still debated, and controversial results have been obtained by both preclinical and clinical studies. Some PXR gene variants have been correlated with NAFLD severity and could be responsible for the progression towards more severe disease stages [62]. Several studies demonstrated that a significant activation of PXR in mice fed with a diet rich in fat and cholesterol promoted the typical hallmarks of NAFLD and NASH, such as steatosis, inflammation, and lipotoxicity, suggesting that PXR activation seems to increase lipid accumulation in the liver, promoting a “fatty phenotype” [63,64,65]. Consistently, a reduction in high-fat diet (HFD)-induced obesity was observed in PXR KO mice and was correlated with an upregulation of FGF15 expression, which suppresses the synthesis of bile acids and reduces lipid absorption and triglycerides in the liver [66]. Accordingly, PXR resulted in being upregulated in the pediatric patients enrolled in the TONIC trial, affected by fibrosis associated with NAFLD. Based on the prognostic relevance of fibrosis in NAFLD patients, PXR has been suggested to have a relevant clinical implication [67,68]. A transcriptome analysis revealed a significant difference between mice fed with HFD and mice fed with a low-fat diet [69]. In detail, Xilin and collaborators analyzed PXR and CAR-modulated CYP3A expression and activity, observing that the administration of HFD activated PXR, inducing both the expression and enzymatic activity of CYP3A11, the murine homologue of the human CYP3A4 [70]. The strong correlation between CYP3A11 expression and PXR activation was observed in all the stages of the disease, from the uncomplicated steatosis to the development of NASH and liver fibrosis [69]. This transcriptional effect on CYP3A11 was not related to CAR activity. Additionally, the activation of PXR regulates specific transcription factors involved in the metabolic homeostasis of the liver, in particular the sterol regulatory element binding protein (SREBP1) and PPARα [71]. Hence, PPARα is involved in lipid metabolism and translocation, fatty acid (FA) oxidation, and glucose synthesis [72,73]. Patients and mice with NAFLD showed reduced levels of hepatic PPARα with respect to those of PPARδ, leading to an increased lipogenesis and decreased fatty acid oxidation [71]. In conclusion, according to these studies, PXR activation induces steatosis in the liver by a complex mechanism leading to both an increase in lipogenesis and a decrease in the β-oxidation of FAs [71,74]. 

However, PPARα activation is also responsible for the production of fibroblast growth factor 21 (FGF21) [75], whose signaling plays a key role in the progression of NAFLD, due to a direct crosstalk with PXR and CYP3A4-related pathways [63,71]. Many studies indicated that the expression and activity of CYP3A4 decrease with the progression of steatosis, probably following the FGF21–PXR–CYP3A4 axis [36,63,76]. The relevance of CYP3A4 modulation is linked to its prominent role in the metabolism of drugs since more than 50% of the drugs currently used are metabolized by this enzyme [77,78]. As demonstrated by the interesting findings of Woolsey and collaborators, NAFLD is characterized by an excess of endoplasmic reticulum (ER) stress that induces the expression of FGF21. This growth factor might enhance mitogen-activated protein kinase (MAPK), which phosphorylates PXR, modulating its intracellular localization. This process results in a reduction in PXR translocation in the nucleus, subsequently leading to a decreased PXR-mediated activation of CYP3A4 transcription [76]. Additionally, in vitro experiments demonstrated that changes in CYP3A4 expression might be associated with free FA-induced steatosis [79].

One of the reasons for the controversial data that have been obtained could reside in the difference in the mechanisms observed in preclinical models and humans. For example, it has been demonstrated that the activation of PXR transcriptional activity leads to opposite effects on gluconeogenesis in rodents and humans [64]. Moreover, the possible influence of disease severity on PXR activation and role can be an explanation of the observed inconsistencies.

In conclusion, more studies are needed to unravel whether PXR plays a role in the development and the progression of NAFLD/NASH and to understand the timing of its function in the different stages of this complex disease.

## 5. PXR and Liver Fibrosis

Liver fibrosis is the main outcome of chronic liver diseases of different etiologies, such as excessive consumption of alcohol (ALD), viral hepatitis (HBV, HCV), steatosis, and cholestasis [80]. Mechanistically, fibrosis is the result of an impaired regenerative response to liver injury that leads to abnormal wound-healing processes, characterized by an accumulation of extracellular matrix (ECM) and inflammatory mediators [10,80]. The main cellular features of this condition are hepatocyte damage, recruitment of inflammatory cells from circulation to the damaged site, and activation of the collagen-producing hepatic stellate cells (HSCs) [81].

Liver fibrosis represents a condition of liver damage affecting more than 800 million people worldwide [82]. Although reversible, fibrosis could progress to life-threatening conditions such as liver cirrhosis and HCC [80]. Fibrogenesis is regulated by the fine crosstalk between several liver cells, such as Kupffer cells, damaged hepatocytes, activated HSCs, monocyte-derived macrophages (MomFs), and other immune cells. In consequence of liver damage, several stimuli activate Kupffer cells, which produce interleukins (IL-6, IL-1β), chemokines (CCL5), growth factors (TGFβ, PDGF), and reactive oxygen species (ROS) that activate hepatic stellate cells (HSCs) and recruit inflammatory MomFs [82,83]. The growth factor TGF-β1 is responsible for the phenotypic change of quiescent HSCs to myofibroblasts, able to secrete excessive amounts of ECM [80]. Furthermore, it is known that in patients with chronic liver disease, a particular subtype of MomFs (CD14highCD16+) is responsible for the production of the pro-inflammatory cytokines TNFα and IL-1β, which also activate HSCs and contribute to disease progression [83].

The expression of PXR has been described in HSCs, monocytes, and resident macrophages, including Kupffer cells [84,85]. Many studies on animal models of liver fibrosis and human specimens have been undertaken to unravel the possible role of PXR as an antifibrotic factor (Table 3). In carbon tetrachloride (CCl_4_)-induced fibrosis, the PXR activator PCN is able to decrease fibrogenesis by downregulating the TGF–β1 pathway and reducing HSC activation [84,86]. These findings have also been confirmed by experiments on human myofibroblasts, which demonstrated that the treatment with PXR activators leads to the inhibition of HSC differentiation to fibroblasts [10]. Similar effects have been demonstrated in other experimental models, such as animals treated with thioacetamide or fed with a high-fat–cholesterol (HFC) diet, where a reduction in PXR expression was observed with respect to controls. Accordingly, the treatment with PXR activators reduced fibrosis and improved liver functions (see [12] and references therein).

These observations have also been essentially confirmed in humans since a decrease in PXR expression in the liver of patients with severe fibrosis was observed regardless of the etiology (ALD, HCV, and PSC), which has been associated with a reduced metabolic capacity of the liver and hepatic failure [87,88,89]. Another pro-inflammatory factor playing a key role in fibrogenesis is NF-kB. Indeed, the NF-kB pathway enhances TGF-β signaling by downregulating the TGF-β receptor BAMBI, thereby triggering the differentiation of HSCs [90]. As already explained, the PXR-mediated inhibition of NF-kB was demonstrated by in vivo studies on rodent models of IBD, showing that PXR is able to inhibit the inflammatory cascade triggered by NF-kB [10]. Wallace and colleagues confirmed these findings also in a mouse model of liver fibrosis, reporting that PCN reduces hepatic NF-kB activity, inflammation, and fibrosis [10]. PXR also causes the enhancement of hepatocyte growth, which is a fundamental feature of liver regeneration after an injury. The activation of PXR regulates cell proliferation, preventing the transcription of cell-cycle suppressors. Therefore, PXR activation promotes the proliferation of hepatocytes by accelerating their cell cycle [19].

## 6. PXR and Liver Cancer

### 6.1. PXR and Hepatocellular Carcinoma

Hepatocellular carcinoma (HCC), the most common primary tumor affecting the liver, represents the third cause of cancer-related death in the world. HCC onset and progression can be summarized by the description of the different phases characterizing its development. The “initiation phase” consists in DNA damage, due to different causes including exposure to chemical compounds. This leads to aberrant alterations of oncogenes and tumor suppressor genes. In the “promotion phase”, hepatocytes display a high proliferation rate, while in the last phase, i.e., the “progression phase”, hepatic cells progress to a neoplastic phenotype, sustaining tumor progression and uncontrolled growth [13].

The pleiotropic effects of PXR in cancer have not been completely unraveled, and controversial observations are reported in the literature in this regard (see [1], Table 4). Kotiya and collaborators observed that the PXR gene and protein levels were reduced in a mouse model of HCC obtained by DEN treatment, whereas the expression of the two pro-inflammatory cytokines IL-6 and TNFα was upregulated. Moreover, the stable transfection of HepG2 cell line with PXR reduced cell migration, adhesion, and invasion, thus suggesting that high PXR levels may play an antitumoral role in the liver [91]. At variance, many studies have demonstrated a correlation between PXR activation and hepatocyte proliferation, which could prompt HCC development. In this regard, Yoshinari and colleagues compared mice treated with the PXR activator PCN with mice treated with PCN in combination with the constitutive androstane receptor (CAR) ligand 1,4-bis-[2-(3,5-dichloropyridyloxy)]-benzene (TCPOBOP) [13]. CAR is another transcription factor known to have a crosstalk with PXR and to be activated by xenobiotics, such as phenobarbital (PB) [13,92]. The study demonstrated that PXR activation is not able to induce cancerous hepatocytes proliferation without the concomitant activation of CAR, indicating that both receptors are needed for HCC development [13]. According to these authors, PXR enhances the transcriptional activity of CAR, modulating the cell cycle since its activation reduces the expression of cell-cycle suppressor genes (*Cdkn1b*, *Rbl2*, *Cdkn1a*), accelerating tumor progression and prompting hepatocytes proliferation. Other in vivo experiments confirm the crosstalk between PXR and other cell-cycle regulators, such as forkhead box proteins (FOXO). In summary, exposure to xenobiotics as environmental pollutants and toxins induces PXR and CAR activation and the consequent hyperproliferation of hepatocytes, thus prompting cancer development. This process is further sustained by PXR activation, by downregulating FOXO-dependent suppressor genes, with an impact on cell cycle [93]. The mutual role of PXR and CAR in hepatic carcinogenesis has been confirmed by Shizu and colleagues, who conducted in vivo experiments with PCN and phenobarbital and demonstrated that PXR activation increases the proliferation of hepatocytes and becomes carcinogenic only when PXR activation is concomitant with CAR stimulation [94]. In another study, the same authors analyzed the effect of long-term PXR activation with PCN in a mouse model of liver cancer induced by DEN, observing no PCN effect on cancer progression. Nevertheless, a 22-week co-treatment with PCN and PB increased the expression of proliferation markers and the number of preneoplastic foci [94]. In contrast, a more prolonged PXR activation (35 week) attenuated CAR-mediated liver cancer promotion, by inhibiting the epithelial–mesenchymal transition (EMT) of hepatocytes. Thus, the authors suggest that the complex interplay between CAR and PXR should be further exploited to become a pharmacological target for cancer chemotherapy.

Another interesting role of PXR is linked to resistance to cancer chemotherapy. Indeed, it has been demonstrated that PXR is able to contribute to chemotherapy resistance in many types of cancer, e.g., colon, breast, and prostate cancer, since its activation regulates transcription of chemoresistance-related genes, such as drug-metabolizing enzymes (e.g., CYP3A4) and transporters (P-glycoprotein and other multidrug resistance proteins) [95,96]. Both preclinical in vitro and clinical studies showed that PXR upregulation decreased the efficacy of the anticancer drugs sorafenib and doxorubicin by a mechanism known as TGF-β-induced chemoresistance in liver cancer [97]. Indeed, TGF-β prompts the non-canonical ERK signaling pathway, activating the transcription factor ETS1 that binds to the PXR promoter, promoting its transcription. This TGF-β/ERK/ETS1/PXR cascade leads to increased expression of efflux transporters and drug metabolizing enzymes, thereby inducing drug resistance [97].

### 6.2. PXR and Cholangiocarcinoma

Cholangiocarcinoma (CCA) is a malignancy of the bile duct classified according to the affected anatomical areas in distal CCA (dCCA), intrahepatic CCA (iCCA), and perihilar CCA (pCCA) [98]. In detail, iCCA is situated near the second-order bile ducts, while perihilar and distal CCA are located in the distal extremities of the liver. Since pCCA originates from the left or right hepatic duct and dCCA interests the bile duct, they might be considered as extrahepatic CCAs (eCCA) [99]. These malignancies are often characterized by high and variable mortality rates, depending on gender, genetic mutations, and geographical origins of the patients, since they more severely affect patients of the Eastern countries [98,100]. Interestingly, CCA has been found to be widely spread in the northeastern region of Thailand, hypothesizing that *Opisthorchis viverrini* infection might represent an important risk factor for the development of this cancer [100]. PSC represents another risk factor because biliary inflammation and bile flow impairment lead to an overexposure of cholangiocytes to toxic BAs, causing a progressive destruction of intrahepatic bile ducts [101]. Chronic viral diseases such as hepatitis B and C can turn into liver cirrhosis, commonly characterized by an insufficient bile acid excretion and an increase in proinflammatory factors, attracting additional bacterial contaminants to the biliary traits [102]. Furthermore, it has also been demonstrated that metabolic dysfunctions, including type I and II diabetes and NAFLD, increase the risk of both intra and extrahepatic CCA. The proposed mechanism relies on the fact that an excess of adipose tissue contributes to insulin resistance and type II diabetes, leading to a compensatory hyperinsulinemia and an enhanced production of the insulin-growth factor-1 (IGF-1), whose signaling pathway increases cell proliferation. To strengthen this association, a study performed by Alvaro and colleagues found that CCA patients express significantly higher levels of IGF-1 compared with healthy livers [102]. Hepatic inflammation and cirrhosis of NAFLD patients have been associated with CCA development since it has been demonstrated that an increase in pro-inflammatory signaling molecules induces the growth of cholangiocytes and DNA damage [103].

Regardless of the etiology, CCA is characterized by a severe transformation of cholangiocytes, together with high concentrations of conjugated BAs in the liver and in the blood [100,104]. It has been hypothesized that conjugated BAs may play a key role in the development of CCA since the bile of CCA patients was found to be enriched of taurine and glycine-conjugated BAs [101]. This hypothesis has been strengthened by in vitro and in vivo studies, respectively conducted on QBC939 CCA cells and xenograft mouse models, demonstrating that conjugated BAs were able to increase the growth of tumoral cells, whereas free BAs exert beneficial roles by inhibiting tumor development [101].

Despite the well-known relationship between BAs and NRs, which have been described in detail above, only a few studies attempted to address this issue in CCA. The role of the PXR partner RXRα has been investigated by Huang and colleagues, who reported its overexpression in the cell cytoplasm of human CCA tissues and in CCA cell lines. For the first time, a role for RXRα has been proposed in CCA growth, by the activation of the Wtn/β-catenin pathway, enhancing the activity of the cell-cycle regulator cyclin D1. Moreover, it has been demonstrated that the knockdown of RXRα decreased the survival of CCA cells by reducing the expression of two G1/S regulators, such as cyclin D1 and E, leading to cell-cycle arrest [105]. Thus, targeting RXRα might be a strategy for the development of new therapeutic agents against CCA.

The NR FXR might also be a promising pharmacological target for CCA therapy. Dai and colleagues conducted several in vitro and in vivo experiments, demonstrating that CCA cells treated with unconjugated BAs—namely, CA, DCA, and CDCA—enhanced the expression of FXR, while conjugated BAs decreased its expression. In addition, CCA cell proliferation was significantly reduced after treatment with unconjugated BAs, so that FXR expression was inversely correlated with cell growth. Additionally, they demonstrated that the FXR agonist GW4064 decreased cell viability and abrogated the effect of pro-carcinogenic conjugated BAs [106].

Considering these findings on the involvement of RXRα-, which dimerizes with PXR to promote gene transcription, and FXR-mediated pathways in CCA, the putative role of PXR should also be evaluated in this hepatic malignancy.

## 7. PXR-Related Gender Differences in Chronic Liver Disease

A marked gender difference characterizes chronic liver diseases since males are more prone to develop NAFLD/NASH, fibrosis, and HCC with respect to females [107]. Accordingly, sex-related dimorphisms of hepatic PXR have been postulated and investigated in many studies. Baretto and colleagues demonstrated that PXR has a female-specific inhibitory activity on the transcription of hepatic genes involved in immune functions such as cytokine secretion and leukocyte recruitment [107]. Interestingly, the genetic deletion of PXR downregulates the transcription of these genes more severely in female than male mice. Although the mechanisms underlying this effect remain essentially unknown, it has been demonstrated that the activation of PXR could interfere with the homeostasis of sex hormones by increasing the catabolism of androgen and estrogens operated by CYPs and sulfotransferases. Thus, it has been hypothesized that PXR concurs in maintaining steroid hormone balance, sexual maturation, and hepatic immune function in females. The loss of PXR induced a significant downregulation of hepatic sex-specific genes regulating osteoclast differentiation, T cell activation, phagocytosis, and inflammatory responses [107]. In addition, it has been demonstrated that PXR activation might impact the metabolism of glucose and lipids, as well as xenobiotic detoxification, in a gender-related manner [107]. Hence, PXR-humanized (hPXR) transgenic mice allowed the observation that PXR played a gender-dependent role in ethanol-induced liver damage. Indeed, although the level of alcohol dehydrogenase (ADH) was comparable in female and male mice, females were less affected by ethanol-induced acute injury than males [108]. Accordingly, further analysis evaluating CYP2E1 and lactoperoxidase (LPO) levels, which are the source and the main marker of oxidative stress, respectively, revealed that the amount of oxidative stress was higher in males [108]. In the same model, gender-related differences have also been demonstrated in lipid metabolism since females showed an increased mRNA expression of genes involved in the uptake of fatty acids, in the catabolism of lipids, and in the formation of very low-density lipoproteins (VLDL) (*L-fabp-1* and *Mttp*). On the other hand, male mice had a greater susceptibility to the development of steatosis with respect to females, where an inhibition of the pro-steatotic *Pemt* gene was observed [108].

Moreover, gender-related multiple gene variants or post-translational modifications of PXR structure have been reported in many liver diseases and might be considered as risk factors. For example, it has been demonstrated that single nucleotide polymorphisms (SNPs) within the promoter and intron1 binding sites of the PXR regulatory region lead to an impaired expression of PXR and CYP3A4 and that this has been connected to drug-induced liver damage after antituberculosis therapy [109]. SNPs present in PXR exons are responsible for modifications of the LBD or DBD, affecting the interactions with ligand compounds or DNA, whereas SNPs in the noncoding sequences usually affect transcription, post-transcriptional modifications, and translation [23]. The so called NR1I2-rs7643645 polymorphism is located within the intron1b region, near to the promotor sequence that specifically binds the hepatic nuclear factor 4α (HNF4α). A loss of this binding site has been observed in consequence of the mutation from A to G allele NR1I2-rs7643645 and causes a loss of 82% HNF4α-related genes in males. In females, a suppression of a lower percentage (52%) of transcription has been noticed, accompanied by an upregulation of a small number of HNF4α-related genes [109]. It should be noticed that the lack of HNF4α in the liver seems to be associated with impaired lipid, glucose, cholesterol, and BA homeostasis and is considered as a pivotal regulator of hepatic transcriptome. Some of the genes downregulated in HNF4α-liver knockout mice in a male-specific manner are Cyp7b1, involved in the synthesis of BAs, Hsd3b4 (hydroxysteroid dehydrogenase 4), and Nox4 (NADPH oxidase 4), involved in oxidative stress [110,111].

In summary, although the role of gender in the regulation of PXR and its target genes in chronic liver diseases are far from being completely elucidated, there is evidence that PXR function is differently modulated in males and females and that SNPs in PXR sequence affect the hypofunction of HFN4α in a gender-related manner since the downregulation of sex-specific genes prominently affects males. As explained before, a loss of HFN4α has been widely correlated with liver inflammation, fibrosis, hepatocellular carcinoma, and aberrant changes in physiological functions such as drug metabolism and transport [112]. Further preclinical and clinical studies involving both males and females are encouraged to gain insight into this topic.

## 8. Conclusions

Many in vitro and in vivo studies demonstrated the involvement of PXR in chronic liver diseases of different etiologies, together with its known *xenosensor* function, operated by modulating the expression of drug metabolizing enzymes and drug transporters. It has also been demonstrated that PXR activity is connected with that of other NRs, such as CAR and PPARs, which in turn orchestrate a complex network of responses that, in addition to regulating DMETs, are involved in both liver damage progression and repair, and in the neoplastic transition to HCC [92].

In conclusion, although many pathways of these finely tuned networks still need to be fully understood and characterized, probably due to the species-related differences that can reduce the translational relevance of animal studies and to changes related to the natural history of the diseases, PXR and its modulation could represent a promising pharmacological target for the identification of novel therapeutical approaches for chronic liver diseases.

## Figures and Tables

**Table 1:** Main studies investigating the involvement of PXR in cholestatic liver diseases.

Effect	Model	Reference
Increase in PXR gene and protein levels	Patients with obstructive cholestasis	[28,29]
PXR downregulation in the late stage of obstructive cholestasis	Children with biliary atresia	[30]
PXR upregulation in the first stages and downregulation in the late stages of cholestatic disease	BDL rats	[43]
A SNP the exon 9 of PXR is weakly correlated with the progression and severity of PBC	PBC patients	[47]
Reduction in PXR expression with respect to healthy subjects	PBC patients grade III and IV	[48]
Bezafibrate increases PXR gene expression exerting anticholestatic effect	PBC patients	[49]
Rifampicin-induced PXR activation mediates the upregulation of UGT1A1 and MRP2	Rifampicin-treated patients undergoing cholecystectomy	[55,56]
PXR overexpression correlates with SULT2A1 upregulation only in PBC patients	PBC and PSC patients	[57]

**Table 2:** Main studies investigating the involvement of PXR in NAFLD/NASH.

Effect	Model	Reference
HFD-induced PXR activation promotes steatosis, inflammation, and lipotoxicity	Mice fed with high fat and cholesterol diet	[65]
PXR genetic deletion reduces HFD-induced obesity and FGF15 expression, decreases the absorption of BAs and lipids and hepatic triglycerides	HFD-fed PXR KO mice	[66]
PXR expression is upregulated according to the degree of fibrosis	Pediatric and adult patients with NAFLD and fibrosis	[67,68]
HFD induces PXR upregulation and increases CYP3A11 expression and activity	HFD-fed mice	[69]
PXR regulates the expression of SREBP1 and PPARα, increases lipogenesis and reduces the oxidation of fatty acids	NAFLD mice and patients review	[74]
HFD reduces PXR activation and CYP3A4 expression due to an imbalance of the FGF21–PXR–CYP3A4 axis	Lipid-loaded Huh7 cells and HFD-fed mice	[76]

**Table 3:** Main studies investigating the involvement of PXR in liver fibrosis.

Effect	Model	Reference
PXR activators inhibit HSC differentiation and reduce hepatic inflammation and fibrosis	Human myofibroblasts and SJL/J mice	[10]
Decrease in PXR expression	Mice treated with thioacetamide or fed with a high-fat–cholesterol (HFC) diet	[12]
PCN-induced PXR activation decreases fibrogenesis by downregulating TGF–β1 pathway and HSC transdifferentiation	Mice with CCl_4_-induced fibrosis	[86]
Decrease in PXR expression in end-stage liver disease, correlated with the downregulation of GSTA1 and CAR	Patients with end-stage liver disease of various etiologies	[87]
Decrease in PXR expression	Patients with viral hepatitis and fibrosis	[88,89]

**Table 4:** Main studies investigating the involvement of PXR in liver cancer (HCC).

Effect	Model	Reference
Decreased PXR gene and protein expression, IL6 and TNFα upregulation PXR transfection reduces HepG2 migration, adhesion, and invasion	DEN-treated mice PXR-transfected HepG2 cells	[91]
PXR enhances the transcriptional activity of CAR and downregulates cell-cycle suppressor genes.	PCN-treated mice	[93]
PXR activation increases hepatocytes proliferation but does not affect cancer progression, whereas concomitant PXR and CAR activation has a pro-carcinogenic effect	Mice with DEN-induced HCC treated with PCN and phenobarbital	[94]
The TGF-β/ERK/ETS1/PXR cascade upregulates efflux transporters and drug metabolizing enzymes, inducing drug resistance	HepG2, Huh7, and PLC/PRF/5 cells	[97]

## References

[1] Xing Y., Yan J., Niu Y. (2020). PXR: A Center of Transcriptional Regulation in Cancer. Acta Pharm. Sin. B.

[2] Weikum E.R., Liu X., Ortlund E.A. (2018). The Nuclear Receptor Superfamily: A Structural Perspective: The Nuclear Receptor Superfamily. Protein Sci..

[3] Papageorgiou L., Shalzi L., Pierouli K., Papakonstantinou E., Manias S., Dragoumani K., Nicolaides N., Giannakakis A., Bacopoulou F., Chrousos G. (2021). An Updated Evolutionary Study of the Nuclear Receptor Protein Family. World Acad. Sci. J..

[4] Mackowiak B., Wang H. (2016). Mechanisms of Xenobiotic Receptor Activation: Direct vs. Indirect. Biochim. Biophys. Acta Gene Regul. Mech..

[5] Motta S., Callea L., Giani Tagliabue S., Bonati L. (2018). Exploring the PXR Ligand Binding Mechanism with Advanced Molecular Dynamics Methods. Sci. Rep..

[6] Mukha A., Kalkhoven E., van Mil S.W.C. (2021). Splice Variants of Metabolic Nuclear Receptors: Relevance for Metabolic Disease and Therapeutic Targeting. Biochim. Biophys. Acta Mol. Basis Dis..

[7] Pavek P. (2016). Pregnane X Receptor (PXR)-Mediated Gene Repression and Cross-Talk of PXR with Other Nuclear Receptors via Coactivator Interactions. Front. Pharmacol..

[8] Li Z., Kruijt J.K., der Sluis R.J.v., Berkel T.J.C.V., Hoekstra M. (2013). Nuclear Receptor Atlas of Female Mouse Liver Parenchymal, Endothelial, and Kupffer Cells. Physiol. Genom..

[9] Lu P., Prost S., Caldwell H., Tugwood J.D., Betton G.R., Harrison D.J. (2007). Microarray Analysis of Gene Expression of Mouse Hepatocytes of Different Ploidy. Mamm Genome.

[10] Wallace K., Cowie D.E., Konstantinou D.K., Hill S.J., Tjelle T.E., Axon A., Koruth M., White S.A., Carlsen H., Mann D.A. (2010). The PXR Is a Drug Target for Chronic Inflammatory Liver Disease. J. Steroid Biochem. Mol. Biol..

[11] Haughton E.L., Tucker S.J., Marek C.J., Durward E., Leel V., Bascal Z., Monaghan T., Koruth M., Collie–Duguid E., Mann D.A. (2006). Pregnane X Receptor Activators Inhibit Human Hepatic Stellate Cell Transdifferentiation In Vitro. Gastroenterology.

[12] Daujat-Chavanieu M., Gerbal-Chaloin S. (2020). Regulation of CAR and PXR Expression in Health and Disease. Cells.

[13] Yoshinari K. (2019). Role of Nuclear Receptors PXR and CAR in Xenobiotic-Induced Hepatocyte Proliferation and Chemical Carcinogenesis. Biol. Pharm. Bull..

[14] Chai S.C., Wright W.C., Chen T. (2020). Strategies for Developing Pregnane X Receptor Antagonists: Implications from Metabolism to Cancer. Med. Res. Rev..

[15] Lamba V. (2004). PXR (NR1I2): Splice Variants in Human Tissues, Including Brain, and Identification of Neurosteroids and Nicotine as PXR Activators. Toxicol. Appl. Pharmacol..

[16] Breuker C., Planque C., Rajabi F., Nault J.-C., Couchy G., Zucman-Rossi J., Evrard A., Kantar J., Chevet E., Bioulac-Sage P. (2014). Characterization of a Novel PXR Isoform with Potential Dominant-Negative Properties. J. Hepatol..

[17] Matic M., Corradin A.P., Tsoli M., Clarke S.J., Polly P., Robertson G.R. (2010). The Alternatively Spliced Murine Pregnane X Receptor Isoform, MPXRΔ171–211 Exhibits a Repressive Action. Int. J. Biochem. Cell Biol..

[18] Hall A., Chanteux H., Ménochet K., Ledecq M., Schulze M.S.E.D. (2021). Designing out PXR Activity on Drug Discovery Projects: A Review of Structure-Based Methods, Empirical and Computational Approaches. J. Med. Chem..

[19] Oladimeji P.O., Chen T. (2018). PXR: More Than Just a Master Xenobiotic Receptor. Mol. Pharmacol..

[20] Halgren T.A. (2009). Identifying and Characterizing Binding Sites and Assessing Druggability. J. Chem. Inf. Modeling.

[21] Cai X., Young G.M., Xie W. (2021). The Xenobiotic Receptors PXR and CAR in Liver Physiology, an Update. Biochim. Et Biophys. Acta Mol. Basis Dis..

[22] Creamer B.A., Sloan S.N.B., Dennis J.F., Rogers R., Spencer S., Mccuen A., Persaud P., Staudinger J.L. (2020). Cancer Growth and Progression. Cells.

[23] Mbatchi L.C., Brouillet J.-P., Evrard A. (2018). Genetic Variations of the Xenoreceptors NR1I2 and NR1I3 and Their Effect on Drug Disposition and Response Variability. Pharmacogenomics.

[24] Wang L., Lonard D.M., O’Malley B.W. (2016). The Role of Steroid Receptor Coactivators in Hormone Dependent Cancers and Their Potential as Therapeutic Targets. Horm. Canc..

[25] Yan J., Xie W. (2016). A Brief History of the Discovery of PXR and CAR as Xenobiotic Receptors. Acta Pharm. Sin. B.

[26] Fejfar T., Vaňásek T., Hůlek P. (2020). Chronic Cholestatic Liver Diseases—Primary Biliary Cholangitis and Primary Sclerosing Cholangitis. Vnitr. Lek..

[27] Wagner M., Fickert P. (2020). Drug Therapies for Chronic Cholestatic Liver Diseases. Annu. Rev. Pharmacol. Toxicol..

[28] Chai J., Luo D., Wu X., Wang H., He Y., Li Q., Zhang Y., Chen L., Peng Z.-H., Xiao T. (2011). Changes of Organic Anion Transporter MRP4 and Related Nuclear Receptors in Human Obstructive Cholestasis. J. Gastrointest. Surg..

[29] Chai J., Feng X., Zhang L., Chen S., Cheng Y., He X., Yang Y., He Y., Wang H., Wang R. (2015). Hepatic Expression of Detoxification Enzymes Is Decreased in Human Obstructive Cholestasis Due to Gallstone Biliary Obstruction. PLoS ONE.

[30] Chen H.-L., Liu Y.-J., Chen H.-L., Wu S.-H., Ni Y.-H., Ho M.-C., Lai H.-S., Hsu W.-M., Hsu H.-Y., Tseng H.-C. (2008). Expression of Hepatocyte Transporters and Nuclear Receptors in Children With Early and Late-Stage Biliary Atresia. Pediatr. Res..

[31] Goldstein J., Levy C. (2018). Novel and Emerging Therapies for Cholestatic Liver Diseases. Liver Int..

[32] Sultana H., Komai M., Shirakawa H. (2021). The Role of Vitamin K in Cholestatic Liver Disease. Nutrients.

[33] Juřica J., Dovrtělová G., Nosková K., Zendulka O. (2016). Bile Acids, Nuclear Receptors and Cytochrome P450. Physiol. Res..

[34] Jia W., Wei M., Rajani C., Zheng X. (2021). Targeting the Alternative Bile Acid Synthetic Pathway for Metabolic Diseases. Protein Cell.

[35] Jia W., Xie G., Jia W. (2018). Bile Acid–Microbiota Crosstalk in Gastrointestinal Inflammation and Carcinogenesis. Nat. Rev. Gastroenterol. Hepatol..

[36] Gabbia D., Roverso M., Guido M., Sacchi D., Scaffidi M., Carrara M., Orso G., Russo F.P., Floreani A., Bogialli S. (2019). Western Diet-Induced Metabolic Alterations Affect Circulating Markers of Liver Function before the Development of Steatosis. Nutrients.

[37] McGlone E.R., Bloom S.R. (2019). Bile Acids and the Metabolic Syndrome. Ann. Clin. Biochem..

[38] Pandak W.M., Kakiyama G. (2019). The Acidic Pathway of Bile Acid Synthesis: Not Just an Alternative Pathway. Liver Res..

[39] Gulamhusein A.F., Hirschfield G.M. (2020). Primary Biliary Cholangitis: Pathogenesis and Therapeutic Opportunities. Nat. Rev. Gastroenterol. Hepatol..

[40] Staudinger J.L., Goodwin B., Jones S.A., Hawkins-Brown D., MacKenzie K.I., LaTour A., Liu Y., Klaassen C.D., Brown K.K., Reinhard J. (2001). The Nuclear Receptor PXR Is a Lithocholic Acid Sensor That Protects against Liver Toxicity. Proc. Natl. Acad. Sci. USA.

[41] Copple B.L., Li T. (2016). Pharmacology of Bile Acid Receptors: Evolution of Bile Acids from Simple Detergents to Complex Signaling Molecules. Pharmacol. Res..

[42] Memon N., Griffin I.J., Lee C.W., Herdt A., Weinberger B.I., Hegyi T., Carayannopoulos M.O., Aleksunes L.M., Guo G.L. (2020). Developmental Regulation of the Gut–Liver (FGF19-CYP7A1) Axis in Neonates. J. Matern. Fetal Neonatal. Med..

[43] Gabbia D., Pozza A.D., Albertoni L., Lazzari R., Zigiotto G., Carrara M., Baldo V., Baldovin T., Floreani A., De Martin S. (2017). Pregnane X Receptor and Constitutive Androstane Receptor Modulate Differently CYP3A-Mediated Metabolism in Early- and Late-Stage Cholestasis. World J. Gastroenterol..

[44] Chang J.-C., Go S., de Waart D.R., Munoz-Garrido P., Beuers U., Paulusma C.C., Oude Elferink R. (2016). Soluble Adenylyl Cyclase Regulates Bile Salt-Induced Apoptosis in Human Cholangiocytes: Autoimmune, Cholestatic and Biliary Disease. Hepatology.

[45] Karlsen T.H., Folseraas T., Thorburn D., Vesterhus M. (2017). Primary Sclerosing Cholangitis—A Comprehensive Review. Hepatology.

[46] Cazzagon N., Sarcognato S., Floreani A., Corrà G., De Martin S., Guzzardo V., Russo F.P., Guido M. (2021). Cholangiocyte Senescence in Primary Sclerosing Cholangitis Is Associated with Disease Severity and Prognosis. JHEP Rep..

[47] Poupon R., Ping C., Chrétien Y., Corpechot C., Chazouillères O., Simon T., Heath S.C., Matsuda F., Poupon R.E., Housset C. (2008). Genetic Factors of Susceptibility and of Severity in Primary Biliary Cirrhosis. Hepatology.

[48] Zollner G., Wagner M., Fickert P., Silbert D., Gumhold J., Zatloukal K., Denk H., Trauner M. (2007). Expression of Bile Acid Synthesis and Detoxification Enzymes and the Alternative Bile Acid Efflux Pump MRP4 in Patients with Primary Biliary Cirrhosis. Liver Int..

[49] Honda A., Ikegami T., Nakamuta M., Miyazaki T., Iwamoto J., Hirayama T., Saito Y., Takikawa H., Imawari M., Matsuzaki Y. (2013). Anticholestatic Effects of Bezafibrate in Patients with Primary Biliary Cirrhosis Treated with Ursodeoxycholic Acid. Hepatology.

[50] Floreani A., De Martin S., Secchi M.F., Cazzagon N. (2019). Extrahepatic Autoimmunity in Autoimmune Liver Disease. Eur. J. Intern. Med..

[51] Etherington R.E., Millar B.J.M., Innes B.A., Jones D.E.J., Kirby J.A., Brain J.G. (2019). Bile Acid Receptor Agonists in Primary Biliary Cholangitis: Regulation of the Cholangiocyte Secretome and Downstream T Cell Differentiation. FASEB Bioadv..

[52] Ronca V., Mancuso C., Milani C., Carbone M., Oo Y.H., Invernizzi P. (2020). Immune System and Cholangiocytes: A Puzzling Affair in Primary Biliary Cholangitis. J. Leukoc. Biol..

[53] Huang K., Mukherjee S., DesMarais V., Albanese J.M., Rafti E., Draghi II A., Maher L.A., Khanna K.M., Mani S., Matson A.P. (2018). Targeting the PXR–TLR4 Signaling Pathway to Reduce Intestinal Inflammation in an Experimental Model of Necrotizing Enterocolitis. Pediatr. Res..

[54] Floreani A., De Martin S. (2021). Treatment of Primary Sclerosing Cholangitis. Dig. Liver Dis..

[55] Xie W., Yeuh M.-F., Radominska-Pandya A., Saini S.P.S., Negishi Y., Bottroff B.S., Cabrera G.Y., Tukey R.H., Evans R.M. (2003). Control of Steroid, Heme, and Carcinogen Metabolism by Nuclear Pregnane X Receptor and Constitutive Androstane Receptor. Proc. Natl. Acad. Sci. USA.

[56] Marschall H., Wagner M., Zollner G., Fickert P., Diczfalusy U., Gumhold J., Silbert D., Fuchsbichler A., Benthin L., Grundstrom R. (2005). Complementary Stimulation of Hepatobiliary Transport and Detoxification Systems by Rifampicin and Ursodeoxycholic Acid in Humans. Gastroenterology.

[57] Wunsch E., Klak M., Wasik U., Milkiewicz M., Blatkiewicz M., Urasinska E., Barbier O., Bielicki D., Bogdanos D.P., Elias E. (2015). Liver Expression of Sulphotransferase 2A1 Enzyme Is Impaired in Patients with Primary Sclerosing Cholangitis: Lack of the Response to Enhanced Expression of PXR. J. Immunol. Res..

[58] Anstee Q.M., Day C.P. (2018). Epidemiology, Natural History, and Evaluation of Nonalcoholic Fatty Liver Disease. Zakim and Boyer’s Hepatology.

[59] Pierantonelli I., Svegliati-Baroni G. (2019). Nonalcoholic Fatty Liver Disease: Basic Pathogenetic Mechanisms in the Progression From NAFLD to NASH. Transplantation.

[60] Gabbia D., Saponaro M., Sarcognato S., Guido M., Ferri N., Carrara M., De Martin S. (2020). Fucus Vesiculosus and Ascophyllum Nodosum Ameliorate Liver Function by Reducing Diet-Induced Steatosis in Rats. Mar. Drugs.

[61] Negi C.K., Bajard L., Kohoutek J., Blaha L. (2021). An Adverse Outcome Pathway Based in Vitro Characterization of Novel Flame Retardants-Induced Hepatic Steatosis. Environ. Pollut..

[62] Sookoian S., Castaño G.O., Burgueño A.L., Gianotti T.F., Rosselli M.S., Pirola C.J. (2010). The Nuclear Receptor PXR Gene Variants Are Associated with Liver Injury in Nonalcoholic Fatty Liver Disease. Pharmacology.

[63] Cobbina E., Akhlaghi F. (2017). Non-Alcoholic Fatty Liver Disease (NAFLD)—Pathogenesis, Classification, and Effect on Drug Metabolizing Enzymes and Transporters. Drug Metab. Rev..

[64] Mackowiak B., Hodge J., Stern S., Wang H. (2018). The Roles of Xenobiotic Receptors: Beyond Chemical Disposition. Drug Metab. Dispos..

[65] Roth A., Looser R., Kaufmann M., Meyer U.A. (2008). Sterol Regulatory Element Binding Protein 1 Interacts with Pregnane X Receptor and Constitutive Androstane Receptor and Represses Their Target Genes. Pharm. Genom..

[66] Zhao L.Y., Xu J.Y., Shi Z., Englert N.A., Zhang S.Y. (2017). Pregnane X Receptor (PXR) Deficiency Improves High Fat Diet-Induced Obesity via Induction of Fibroblast Growth Factor 15 (FGF15) Expression. Biochem. Pharmacol..

[67] Elbel E.E., Lavine J.E., Downes M., Van Natta M., Yu R., Schwimmer J.B., Behling C., Brunt E.M., Tonascia J., Evans R. (2018). Hepatic Nuclear Receptor Expression Associates with Features of Histology in Pediatric Nonalcoholic Fatty Liver Disease. Hepatol. Commun..

[68] Angulo P., Kleiner D.E., Dam-Larsen S., Adams L.A., Bjornsson E.S., Charatcharoenwitthaya P., Mills P.R., Keach J.C., Lafferty H.D., Stahler A. (2015). Liver Fibrosis, but No Other Histologic Features, Is Associated With Long-Term Outcomes of Patients With Nonalcoholic Fatty Liver Disease. Gastroenterology.

[69] Li X., Wang Z., Klaunig J.E. (2018). Modulation of Xenobiotic Nuclear Receptors in High-Fat Diet Induced Non-Alcoholic Fatty Liver Disease. Toxicology.

[70] Taneja G., Maity S., Jiang W., Moorthy B., Coarfa C., Ghose R. (2019). Transcriptomic Profiling Identifies Novel Mechanisms of Transcriptional Regulation of the Cytochrome P450 (Cyp)3a11 Gene. Sci Rep.

[71] Cave M.C., Clair H.B., Hardesty J.E., Falkner K.C., Feng W., Clark B.J., Sidey J., Shi H., Aqel B.A., McClain C.J. (2016). Nuclear Receptors and Nonalcoholic Fatty Liver Disease. Biochim. Et Biophys. Acta Gene Regul. Mech..

[72] Chiazza F., Collino M., Mauricio D. (2016). Chapter 9 Peroxisome Proliferator-Activated Receptors (PPARs) in Glucose Control. Molecular Nutrition and Diabetes.

[73] Vairappan B., Patel V.B. (2016). Chapter 15 Cholesterol Regulation by Leptin in Alcoholic Liver Disease. Molecular Aspects of Alcohol and Nutrition.

[74] Zhou J., Febbraio M., Wada T., Zhai Y., Kuruba R., He J., Lee J.H., Khadem S., Ren S., Li S. (2008). Hepatic Fatty Acid Transporter Cd36 Is a Common Target of LXR, PXR, and PPARγ in Promoting Steatosis. Gastroenterology.

[75] Tezze C., Romanello V., Sandri M. (2019). FGF21 as Modulator of Metabolism in Health and Disease. Front. Physiol..

[76] Woolsey S.J., Beaton M.D., Mansell S.E., Leon-Ponte M., Yu J., Pin C.L., Adams P.C., Kim R.B., Tirona R.G. (2016). A Fibroblast Growth Factor 21-Pregnane X Receptor Pathway Downregulates Hepatic CYP3A4 in Nonalcoholic Fatty Liver Disease. Mol. Pharmacol..

[77] Palatini P., Martin S., Pegoraro P., Orlando R. (2008). Enzyme Inhibition and Induction in Liver Disease. Curr. Clin. Pharmacol..

[78] De Martin S., Gabbia D., Albertin G., Sfriso M.M., Mescoli C., Albertoni L., Paliuri G., Bova S., Palatini P. (2014). Differential Effect of Liver Cirrhosis on the Pregnane X Receptor-Mediated Induction of CYP3A1 and 3A2 in the Rat. Drug Metab. Dispos..

[79] Huang Z., Wang M., Liu L., Peng J., Guo C., Chen X., Huang L., Tan J., Yang G. (2019). Transcriptional Repression of CYP3A4 by Increased MiR-200a-3p and MiR-150-5p Promotes Steatosis in Vitro. Front. Genet..

[80] Aydin M.M., Akcali K.C. (2018). Liver Fibrosis. Turk. J. Gastroenterol..

[81] Gabbia D., Carpi S., Sarcognato S., Cannella L., Colognesi M., Scaffidi M., Polini B., Digiacomo M., Esposito Salsano J., Manera C. (2021). The Extra Virgin Olive Oil Polyphenol Oleocanthal Exerts Antifibrotic Effects in the Liver. Front. Nutr..

[82] Parola M., Pinzani M. (2019). Liver Fibrosis: Pathophysiology, Pathogenetic Targets and Clinical Issues. Mol. Asp. Med..

[83] Wen Y., Lambrecht J., Ju C., Tacke F. (2021). Hepatic Macrophages in Liver Homeostasis and Diseases-Diversity, Plasticity and Therapeutic Opportunities. Cell. Mol. Immunol..

[84] Wright M.C. (2006). The Impact of Pregnane X Receptor Activation on Liver Fibrosis. Biochem. Soc. Trans..

[85] Mohandas S., Vairappan B. (2017). Role of Pregnane X-Receptor in Regulating Bacterial Translocation in Chronic Liver Diseases. World J. Hepatol..

[86] Naito H., Jia X., Yetti H., Yanagiba Y., Tamada H., Kitamori K., Hayashi Y., Wang D., Kato M., Ishii A. (2016). Importance of Detoxifying Enzymes in Differentiating Fibrotic Development between SHRSP5/Dmcr and SHRSP Rats. Environ. Health Prev. Med..

[87] Kurzawski M., Dziedziejko V., Post M., Wójcicki M., Urasińska E., Miętkiewski J., Droździk M. (2012). Expression of Genes Involved in Xenobiotic Metabolism and Transport in End-Stage Liver Disease: Up-Regulation of ABCC4 and CYP1B1. Pharmacol. Rep..

[88] Congiu M., Mashford M.L., Slavin J.L., Desmond P.V. (2009). Coordinate Regulation of Metabolic Enzymes and Transporters by Nuclear Transcription Factors in Human Liver Disease. J. Gastroenterol. Hepatol..

[89] Hanada K., Nakai K., Tanaka H., Suzuki F., Kumada H., Ohno Y., Ozawa S., Ogata H. (2012). Effect of Nuclear Receptor Downregulation on Hepatic Expression of Cytochrome P450 and Transporters in Chronic Hepatitis C in Association with Fibrosis Development. Drug Metab. Pharmacokinet..

[90] Luedde T., Schwabe R.F. (2011). NF-ΚB in the Liver-Linking Injury, Fibrosis and Hepatocellular Carcinoma. Nat. Rev. Gastroenterol. Hepatol..

[91] Kotiya D., Jaiswal B., Ghose S., Kaul R., Datta K., Tyagi R.K. (2016). Role of PXR in Hepatic Cancer: Its Influences on Liver Detoxification Capacity and Cancer Progression. PLoS ONE.

[92] Gabbia D., Pozzo L., Zigiotto G., Roverso M., Sacchi D., Dalla Pozza A., Carrara M., Bogialli S., Floreani A., Guido M. (2018). Dexamethasone Counteracts Hepatic Inflammation and Oxidative Stress in Cholestatic Rats via CAR Activation. PLoS ONE.

[93] Shizu R., Abe T., Benoki S., Takahashi M., Kodama S., Miayata M., Matsuzawa A., Yoshinari K. (2016). PXR Stimulates Growth Factor-Mediated Hepatocyte Proliferation by Cross-Talk with the FOXO Transcription Factor. Biochem. J..

[94] Shizu R., Ishimura M., Nobusawa S., Hosaka T., Sasaki T., Kakizaki S., Yoshinari K. (2021). The Influence of the Long-Term Chemical Activation of the Nuclear Receptor Pregnane X Receptor (PXR) on Liver Carcinogenesis in Mice. Arch. Toxicol..

[95] Qiao E., Ji M., Wu J., Ma R., Zhang X., He Y., Zha Q., Song X., Zhu L.-W., Tang J. (2013). Expression of the PXR Gene in Various Types of Cancer and Drug Resistance. Oncol. Lett..

[96] Cui W., Shen X., Agbas E., Tompkins B., Cameron-Carter H., Staudinger J.L. (2020). Phosphorylation Modulates the Coregulatory Protein Exchange of the Nuclear Receptor Pregnane X Receptor. J. Pharmacol. Exp. Ther..

[97] Bhagyaraj E., Ahuja N., Kumar S., Tiwari D., Gupta S., Nanduri R., Gupta P. (2019). TGF-β Induced Chemoresistance in Liver Cancer Is Modulated by Xenobiotic Nuclear Receptor PXR. Cell Cycle.

[98] Khan S.A., Tavolari S., Brandi G. (2019). Cholangiocarcinoma: Epidemiology and Risk Factors. Liver Int..

[99] Kendall T., Verheij J., Gaudio E., Evert M., Guido M., Goeppert B., Carpino G. (2019). Anatomical, Histomorphological and Molecular Classification of Cholangiocarcinoma. Liver Int..

[100] Myint K., Kongpracha P., Rattanasinganchan P., Leelawat K., Moolthiya P., Chaiyabutr K., Tohtong R. (2017). Gadd45β Silencing Impaired Viability and Metastatic Phenotypes in Cholangiocarcinoma Cells by Modulating the EMT Pathway. Oncol Lett..

[101] Liu R., Zhao R., Zhou X., Liang X., Campbell D.J.W., Zhang X., Zhang L., Shi R., Wang G., Pandak W.M. (2014). Conjugated Bile Acids Promote Cholangiocarcinoma Cell Invasive Growth through Activation of Sphingosine 1-phosphate Receptor 2. Hepatology.

[102] Labib P.L., Goodchild G., Pereira S.P. (2019). Molecular Pathogenesis of Cholangiocarcinoma. BMC Cancer.

[103] Wongjarupong N., Assavapongpaiboon B., Susantitaphong P., Cheungpasitporn W., Treeprasertsuk S., Rerknimitr R., Chaiteerakij R. (2017). Non-Alcoholic Fatty Liver Disease as a Risk Factor for Cholangiocarcinoma: A Systematic Review and Meta-Analysis. BMC Gastroenterol..

[104] Zhou H., Hylemon P.B. (2014). Bile Acids Are Nutrient Signaling Hormones. Steroids.

[105] Huang G., Zhang W., Ren H., Shen X., Chen Q., Shen D. (2015). Retinoid X Receptor α Enhances Human Cholangiocarcinoma Growth through Simultaneous Activation of Wnt/Β-catenin and Nuclear Factor-κB Pathways. Cancer Sci..

[106] Dai J., Wang H., Shi Y., Dong Y., Zhang Y., Wang J. (2011). Impact of Bile Acids on the Growth of Human Cholangiocarcinoma via FXR. J. Hematol. Oncol..

[107] Barretto S.A., Lasserre F., Huillet M., Régnier M., Polizzi A., Lippi Y., Fougerat A., Person E., Bruel S., Bétoulières C. (2021). The Pregnane X Receptor Drives Sexually Dimorphic Hepatic Changes in Lipid and Xenobiotic Metabolism in Response to Gut Microbiota in Mice. Microbiome.

[108] Spruiell K., Gyamfi A.A., Yeyeodu S.T., Richardson R.M., Gonzalez F.J., Gyamfi M.A. (2015). Pregnane X Receptor-Humanized Mice Recapitulate Gender Differences in Ethanol Metabolism but Not Hepatotoxicity. J. Pharmacol. Exp. Ther..

[109] Wang J.Y., Tsai C.H., Lee Y.L., Na Lee L., Hsu C.L., Chang H.C., Chen J.M., Hsu C.A., Yu C.J., Yang P.C. (2015). Gender-Dimorphic Impact of PXR Genotype and Haplotype on Hepatotoxicity During Antituberculosis Treatment. Medicine.

[110] Holloway M.G., Miles G.D., Dombkowski A.A., Waxman D.J. (2008). Liver-Specific Hepatocyte Nuclear Factor-4α Deficiency: Greater Impact on Gene Expression in Male than in Female Mouse Liver. Mol. Endocrinol..

[111] Gabbia D., Cannella L., De Martin S. (2021). The Role of Oxidative Stress in NAFLD–NASH–HCC Transition—Focus on NADPH Oxidases. Biomedicines.

[112] Lu H. (2016). Crosstalk of HNF4α with Extracellular and Intracellular Signaling Pathways in the Regulation of Hepatic Metabolism of Drugs and Lipids. Acta Pharm. Sin. B.

